# Activity of Alzheimer’s γ-secretase is linked to changes of interferon-induced transmembrane proteins (IFITM) in innate immunity

**DOI:** 10.1186/s13024-020-00417-0

**Published:** 2020-11-12

**Authors:** Annie Y. Yao, Riqiang Yan

**Affiliations:** Department of Neuroscience, University of Connecticut Health, Farmington, CT USA

**Keywords:** Γ-secretase, Presenilin, Nicastrin, Interferon-induced transmembrane protein, IFITM3, Innate immunity, Photo-crosslinking, Amyloid plaques, Alzheimer’s disease

## Abstract

The activity of γ-secretase is critical to the pathogenesis of Alzheimer’s disease (AD). How its activity is regulated is intriguing and highly important for any AD therapy that focuses on reduction of toxic amyloid peptides and amyloid deposition in patients. Recently, interferon-induced transmembrane protein 3 (IFITM3) has been identified as a novel regulator of γ-secretase through a specific interaction. This commentary highlights this exciting study and provides an updated link of γ-secretase activity to innate immunity through IFITM3.

Immune activity has long been linked to Alzheimer’s disease (AD) pathology, but whether it is beneficial and detrimental to AD progression has stirred a great deal of debate [7, 12]. The heat of this debate has not ebbed in recent years; rather, the premise of neuroinflammation in AD pathogenesis has become increasingly recognized due to growing evidence that risk genes for late onset of AD are predominantly expressed by microglia [11, 33]. Of the AD risk loci identified by genomic methods, over 50% of validated gene variants are implicated in innate immune and microglial functions, including the top 2 AD risk genes, *APOE* and *TREM2* [14, 36]. Epigenomic analysis shows that AD GWAS loci are preferentially enriched in enhancer sequences involved in innate immune processes [21, 22]. Intriguingly, single cell profiling of human and AD mouse brains has revealed disease associated microglia (DAM) with unique transcriptomic profiles that localize near amyloid plaques [20, 26], while other studies highlight how dysfunctional microglia, with impaired chemotactic and phagocytic functions, result in increased Aβ deposition in the AD brain [1]. These emerging evidence emphasize how microglia, as members of the innate immune system, serve a central role in Alzheimer’s neurodegeneration.

Amyloid-β (Aβ), considered a primary cause of AD, may function as an antimicrobial peptide (AMP) [5, 37], and thus may play a functional role in the innate immune system. Infection from a variety of pathogens, including *Chlamydia pneumoniae* [4, 23], Herpes simplex virus [9], pseudorabies virus [39], *Toxoplasma gondii* [41] and *Porphyromonas gingivalis* [17], leads to increased Aβ production in the brain, suggesting that bacterial and viral infections in human and animal models can shift the processing of amyloid precursor protein (APP) toward the amyloidogenic pathway, resulting in increased rates of Aβ production and deposition. This increased Aβ production is perhaps a defense against invading pathogens. Indeed, Aβ are shown to possess antimicrobial activity against many bacterial and fungal pathogens as well as viruses [13, 27]. The antimicrobial activity of Aβ species may partly be due to its capacity to form fibrils, which can insert into cell membranes to permeabilize microbes, leading to their death. Aβ fibrils can capture and perforate microbes with its hairpin loop, while aggregates of Aβ may immobilize microbes in a manner similar to neutrophil extracellular traps (NETs) [6]. How Aβ production is coordinated in such an elegant immune response remains an outstanding question in the field. Furthermore, the consequences of pathogen-stimulated Aβ production on the risk of developing AD have yet to be fully elucidated.

In a recent study published by the Li lab in *Nature*, an innate defense is shown to stimulate Aβ production, thus increasing AD risk [16]. By using the γ-secretase modulator (GSM) E2012-BPyne, Hur and colleagues conducted photo-crosslinking experiments and identified interferon (IFN)-induced transmembrane protein 3 (IFITM3) as an authentic interaction protein of γ-secretase. IFITM3 is a member of the *IFITM*s, a protein family that was first discovered as interferon-induced genes in human neuroblastoma cells [2, 35]. This family has five members in the human and seven members in the rodent; homologous IFITM members are evolutionally conserved and distinguished by the presence of a canonical CD225 domain, made up of two hydrophobic membrane binding sequences separated by a highly conserved cytosolic loop. This di-spanning CD225 domain resembles the reticulon homolog domain [34], and embeds IFITMs on the lipid bilayer. Likely found on the membrane, IFITM3 interacts with γ-secretase, a protease complex known to have four multi-spanning transmembrane proteins comprised of presenilin-1 (PS1), presenilin enhancer 2, anterior pharynx defective 1, and nicastrin (NCT). Consistent with its original discovery, IFITMs are found to limit virus infection or replication in a variety of vertebrates [2, 24, 32], positing a possible evolutionarily significant role in host immunity across species. IFITMs block viral infection by interacting with nearby IFITMs and/or other transmembrane proteins, thereby reducing host membrane fluidity at the sites of viral fusion and preventing viral fusion pore formation.

Although IFITM proteins can play a known role in innate antiviral and adaptive immunity, virus restriction by individual IFITMs depends on cell type and intracellular localization. For example, IFITM1 is localized on the cell surface and early endosomes, and restricts HIV infection at these sites. Immunofluorescence and live-cell imaging studies reveal cellular localization of IFITM2 and 3 primarily to early and late endocytic vesicles and lysosomes [2, 8, 31]. Studies with influenza A virus in host cells suggest that IFITM2 and 3 restrict the virus entry or fusion at these sites through pH-dependent changes in conformation of envelope glycoproteins [8, 10, 19]. A recent study has shown that IFITM3 directly engages incoming viral particles and enhances the viral trafficking from endosomes to lysosomes [38]. This is consistent with the ability of IFITM3 to limit the entry of a wide range of primarily enveloped viruses into host cells.

Expression of IFITMs is not always dependent on interferon induction, since high levels of constitutively-expressed IFITMs are found in many cell types [2]. Constitutive non-IFN-activated expression of IFITMs maintain persistent antiviral status against a panel of viruses. In human embryonic stem cells, constitutive expression of IFITM3 likely confers intrinsic antiviral activity [43]. IFITMs constitutively expressed by primary CD8^+^ T cells and respiratory dendritic cells may be directly involved in adaptive immunity [42]. This unique expression profile suggests a need to ensure proper function of IFITMs in certain tissues or cell types to counteract pathogenic insults.

In the context of AD, the role of IFITM3 drew focus on an innate immune rather than an adaptive immune process [16]. Hur and colleagues discovered that γ-secretase activity correlates with increased IFITM3 levels. E2012-BPyne-labeled IFITM3 was significantly reduced in PS1 and PS2 double knockout mice compared to wild-type mice. This PS-dependent crosslink of IFITM3 to the γ-secretase complex is not due to a reduction of IFITM3 mRNA levels, but rather more likely related to post-translational stability of the complex.

The authors found that IFITM3 binds directly to the active γ-secretase complex near its active site and reduces γ-secretase activity for the production of Aβ_40_ and Aβ_42_. IFITM3 knockdown by siRNA led to reduced γ-secretase activity, as measured by a reduced production of Aβ_40_ and Aβ_42_. In 5xFAD transgenic mice, which express five familial AD mutations and recapitulate features of AD amyloid pathology [28], IFITM3 levels increased with age, whereas PS1 and NCT levels remained relatively constant. This increase is perhaps related to altered cellular expression profiles and localization: the authors found higher IFITM3 expression in GFAP-labeled astrocytes and IBA1-labeled microglia in the 5xFAD mouse brain as compared to age-matched wild-type littermates, in which IFITM3 expression is primarily detected within the meninges and blood vessels. One may speculate that either gliosis in response to amyloid deposition, or FAD mutations, play a role in the increased glial IFITM3 expression. The authors show that *Ifitm3*^−/−^ mice had comparable γ-secretase complex levels compared to controls, while γ-secretase activity for generating Aβ_40_ and Aβ_42_ was reduced. Consistently, the number of Aβ plaques in 5xFAD/*Ifitm3*^−/−^ transgenic mice was also reduced.

Aging is the biggest risk factor for AD. Aging can also induce type I IFN expression, which can thereby modulate brain function [3]. Viral infection in human brains can alter the cytokine profiles produced by microglia [25]. Not surprisingly, IFNγ has been explored for its role in the pathogenesis of AD through modulation of neuroinflammation [26, 30, 40]. In mouse primary neurons treated with IFNγ or IFNα (a type I IFN), expression of IFITM3 was increased as expected, even as there was no change in the levels of NCT or PS1. Increased IFITM3 levels alone were sufficient to increase the γ-secretase activity as evidenced by higher secreted levels of Aβ_40_ and Aβ_42_. More importantly, elevated IFITM3 levels are evident in a subpopulation of human patients with late-onset Alzheimer’s disease (LOAD), indicating its pathophysiological relevance to AD. Consistent with mouse and in vitro assays, human tissues with high IFITM3 levels also produced more Aβ_40_ and Aβ_42_.

These findings are important, as they directly link Aβ production with innate immunity and neuroinflammation in a novel way. While the prevailing view is that Aβ triggers a cascade of pathological gliosis and neuroinflammation [15, 18], Hur and colleagues have provided compelling evidence of how neuroinflammation may also activate toxic Aβ generation. Of note, it has been previously shown that lipopolysaccharide, prostaglandin E2, and pro-inflammatory cytokines like IL-1β can enhance Aβ accumulation by impairing microglial phagocytosis and clearance of Aβ [29]. However, this paper provides a direct link between Aβ production and aggregation by identifying IFITM3 as a γ-secretase modulatory protein associated with aging and AD. Furthermore, this work is also the first to demonstrate how Aβ has a physiological function as an AMP through the action of IFITM3. Targeting IFITM3 should be explored as an AD therapeutic strategy for reducing amyloid deposition. In particular, the subpopulation of LOAD patients exhibiting high levels of IFITM3 expression and activity, which strongly correlate with γ-secretase activity, may be the most suitable demographic for clinical trials of IFITM3 inhibitors. For the same reason, IFITM3 may well be developed as a biomarker to identify LOAD patients. Taken together, more research on IFITM3 and innate immunity for applications in translational AD therapeutics is warranted. Further study should also focus on optimizing or fine tuning the therapeutic inhibition on IFITM3, in order to avoid undesirable side effects relating to its beneficial effects on innate immunity.

## Conclusion

The activity of γ-secretase can be regulated through the disruption of interaction between IFITM3 and components of γ-secretase complex.

## Data Availability

Not applicable.

## Abbreviations

AD: Alzheimer’s disease
APP: Amyloid precursor protein
Aβ: amyloid-β peptide
AMP: Antimicrobial peptide
DAM: Disease associated microglia
GFAP: Glial fibrillary acidic protein GSM, γ-secretase modulator
IBA: Ionized calcium-binding adaptor molecule 1
IFN: Interferon
IFITM: FAD familial AD, interferon-induced transmembrane protein
LOAD: Late-onset Alzheimer’s disease
NCT: Nicastrin, NETs, neutrophil extracellular traps
PS1: Presenilin 1
